# Carbon Footprint of Antibody‐Based Drugs and Biologics Using Hybrid Life Cycle Assessment

**DOI:** 10.1002/cpt.70301

**Published:** 2026-05-06

**Authors:** Sébastien Taillemite, Martin Fenelon, Marc Scherlinger, Max Piffoux

**Affiliations:** ^1^ Ecovamed Paris France; ^2^ Rheumatology Department, National Reference Center for Rare Autoimmune Systemic Diseases, RESO Strasbourg University Hospital Strasbourg France; ^3^ UMR_S INSERM 1109, Immunorhumatologie Moléculaire Strasbourg France; ^4^ Medical Oncology, Hospices Civils de Lyon Lyon France; ^5^ Medical Oncology Centre Léon Bérard Lyon France

## Abstract

Biological engineering has emerged since the 1980s as one of the most efficient technologies to develop new medicines. The monoclonal antibody platform is the most widely used, representing about 15–20% of drug sales. Only scarce and partial monoclonal antibody life cycle assessments (LCAs) are reported. We estimate the cradle‐to‐pharmacy gate carbon footprint of monoclonal antibodies from the pharmacopeia on the market (*n* = 103) using the full life cycle inventory of the medicines, encompassing antibody production, packaging production, transport, medicine manufacturing, and associated corporate emissions using a hybrid LCA/environmentally extended input–output model. Monoclonal antibody‐based drugs have an important carbon footprint with a mean of 169.7 kgCO_2_e/vial or prefilled syringe (95% CI 10.1–621.1) and 371.4 kgCO_2_e/month (95% CI 23.1–1,479.5) vs. 14.1 kgCO_2_e/month (95% CI 0.33–36.2) for an oral treatment. A large fraction of emissions is emerging from corporate emissions. The 95% CI surrounding these estimations is about ±35%. Among subparts, antibody production emissions largely vary from 7.1 to 20,206 kgCO_2_e/g of antibody (mean 472 kgCO_2_e/g, 95% CI 8.2–2,529), depending on the production scale. As an illustration, the carbon footprint of second‐line treatment options in rheumatoid arthritis ranges from 286 to 2,047 kgCO_2_e/year. In cancer immunotherapy, alternative weight‐adjusted dosing strategies lead to a 10–20% mitigation in greenhouse gas emissions, while “low‐dose” strategies may mitigate emissions by 2‐ to 10‐fold. Antibody‐based drugs have a significant carbon footprint, although highly variable. This database allows for a better understanding of the carbon footprint associated with these drugs, in order to better eco‐design care pathways.

## Graphical abstract

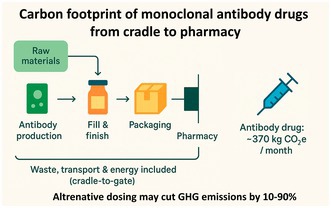

**Study Highlights**

**WHAT IS THE CURRENT KNOWLEDGE ON THE TOPIC?**

Only a few publications, each with a narrow scope, like analyses of single products, specific manufacturing processes, or comparisons of administration routes for individual drugs, are available in the literature.

**WHAT QUESTION DID THIS STUDY ADDRESS?**

The limited evidence suggested that biologic drug production and delivery can be carbon‐intensive, but a full understanding of the magnitude and drivers of emissions across different products was lacking.

**WHAT DOES THIS STUDY ADD TO OUR KNOWLEDGE?**

To our knowledge, this is the first study to comprehensively quantify the carbon footprint of most monoclonal antibodies and related biologics of the pharmacopeia using a hybrid approach that combines process‐based life cycle assessment with an environmentally extended input–output model. We accounted for active pharmaceutical ingredient production, formulation and fill–finish processes, packaging materials, global transport and cold‐chain logistics, as well as corporate‐level emissions associated with research, development, and administration. These drugs are associated with significant emissions, implying that alternative dose schemes are associated with significant gains in greenhouse gas emissions.

**HOW MIGHT THIS CHANGE CLINICAL PHARMACOLOGY OR TRANSLATIONAL SCIENCE?**

This work enlarges the Ecovamed drug carbon footprint database (already containing >12,000 per os drugs and most i.v. drugs). These results can incentivize pharmaceutical innovation aimed at reducing emissions, encouraging alternative drug schemes. By making carbon emissions data available for a major drug class, our work supports more informed, carbon‐conscious choices in healthcare delivery and paves the way for a more sustainable pharmaceutical sector.

Global warming driven by greenhouse gas (GHG) emissions is one of the most significant threats facing humanity, with long‐term and difficult‐to‐reverse consequences. Climate change is projected to cause an estimated 5,500 million excess deaths between 2020 and 2,500.^1^ In this context, healthcare systems will face immense strain, creating a paradoxical feedback loop. Indeed, the healthcare sector accounts for 3–8% of national GHG emissions, with a global average of 5.0–6.5%.^2^, ^3^

Pharmaceuticals constitute a substantial portion of healthcare's carbon footprint, contributing 20–55% of total emissions from health systems.^4^, ^5^, ^6^ However, emission factors derived from the environmentally extended input–output (EEIO) approach lack the necessary granularity to assess individual care pathways. For instance, the US EEIO framework provides only two emission factors for pharmaceuticals—one for oral drugs, including pills, powders and solutions (0.136 kgCO2e/$) and another for vaccines and biological products (0.087 kgCO2e/$).^7^ This limitation hinders efforts to eco‐design care pathways, reduce GHG emissions, and minimize environmental impact at both the procurement and regulatory stages.

The healthcare life cycle assessment (LCA) database^8^ compiles a vast range of medical devices but includes only a limited number of pharmaceuticals.^9^, ^10^, ^11^, ^12^ To address this gap, we recently expanded the literature by publishing the LCA of 12,386 oral drugs.^13^

Biologic therapies, especially monoclonal antibodies (mAbs), antibody drug conjugates (ADCs), and recombinant proteins, now occupy a central place in medical care, accounting for about 20–25% of global pharmaceutical sales in 2022 and an increasing share among the top‐selling medicines.^14^, ^15^ Over three decades, approvals have surged from a handful in the 1990s to nearly >100 new antibody therapeutics entering development each year.^16^ The largest therapeutic markets are oncology and immune‐mediated diseases. Biologics span both rare and common conditions, from orphan indications (e.g., generalized pustular psoriasis; paroxysmal nocturnal hemoglobinuria) to high‐prevalence cancers and autoimmune diseases like rheumatoid arthritis that affects up to 0.5–1% of the population.^17^

In this study, we developed a model to estimate the full LCA of mAbs and other related biologics/biotherapies, with a focus on GHG emissions. Our analysis encompasses production, packaging, fill and finish, transport, and corporate emissions, providing a more comprehensive assessment of their environmental impact.

## METHODS

### Life cycle assessment of monoclonal antibodies drugs

#### Goal and scope definition

The objective of this LCA is to evaluate the carbon footprint of mAbs (and related fusion proteins) on the market, excluding those that are not sold anymore, not sold yet, or those that are only available in a limited number of countries, leaving a total of *n* = 103 antibodies, including biosimilars. The functional unit of this LCA is the supply of one box of medicine to a pharmacy (city or hospital based), with a box being well defined in the database according to its official description giving the name of the active principal ingredient(s) (APIs) or the brand name, the dosage, the pharmaceutical form, the type of primary packaging and the number of units per box. This functional unit is the smallest amount of drug that can be sold, as boxes cannot be opened and divided in most countries. Of note, for a given medicine, different box sizes may exist (e.g., boxes of 1 or 3 vials). Due to the precise description given in the database, the carbon footprint per unit, per daily dose or any other therapeutic scheme can easily be obtained from the carbon footprint per box.

Monoclonal antibody‐based drugs included in the database represented an estimated $183 billion of sales in 2023, based on annual reports and other external communications from pharmaceutical companies which are supplying these 103 medicines, over a total of $220–230 billion in revenues for all mAbs sold in the World in 2023,^14^ so the database represents >80% of the mAb market. Our LCA is performed for the French pharmacopoeia, but the hypothesis used is valid for the European market as an area of consumption. The database is available on www.ecovamed.com.

#### System boundaries

**Figure 1:**
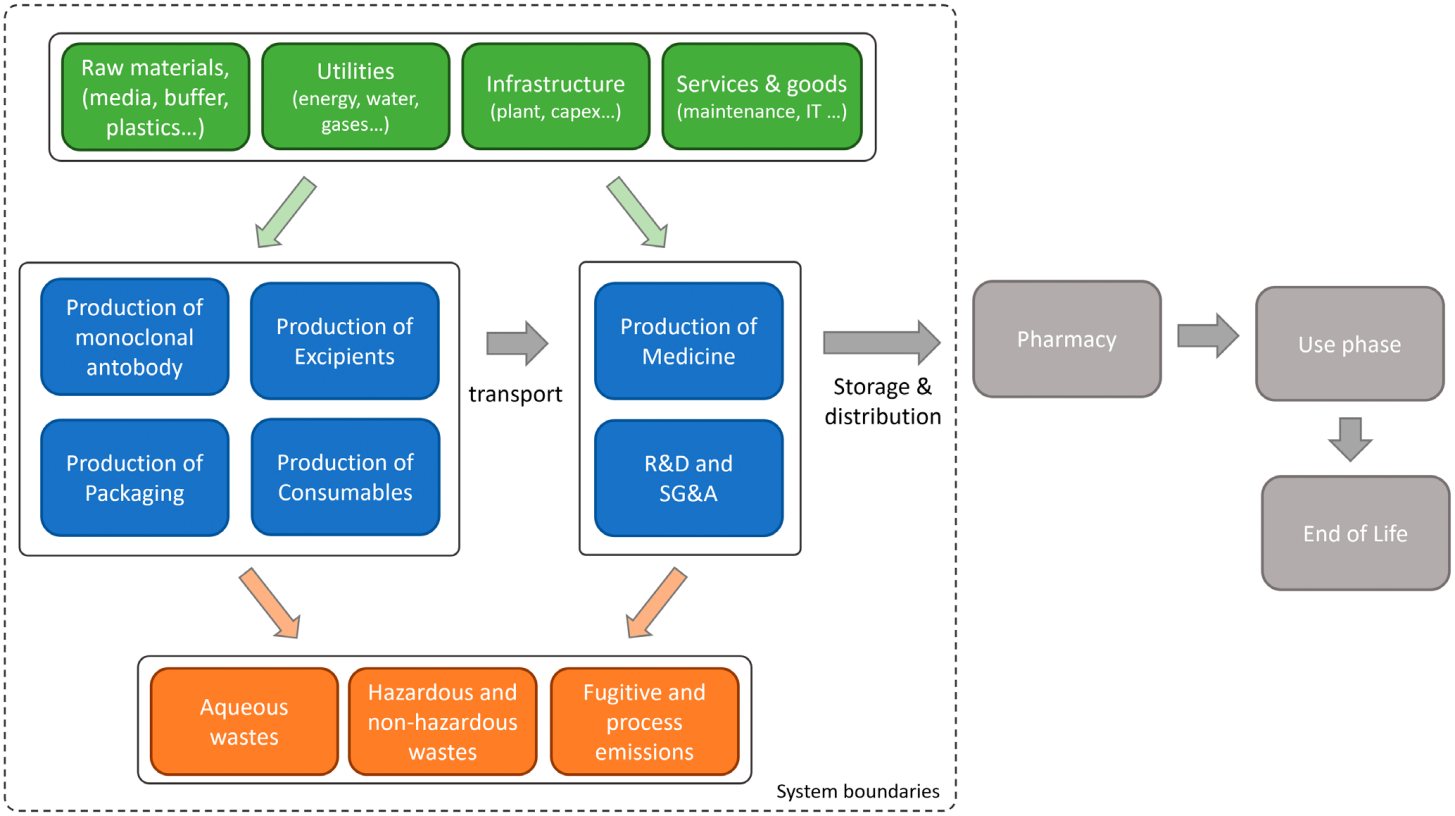
Processes and flows for the supply of one box of medicine to a pharmacy.

The system boundaries include all life cycle stages from material acquisition and pre‐processing, representing the extraction of energy and raw materials, production of the mAb, including the upstream process with mammalian cell culture and the downstream process for purification, transport, packaging, fill, and finish into vials or prefilled syringes, and corporate costs (administration, research and development, sales), to the gate of the pharmacies (**Figure** **1**). The considered processes and flows are energy (electricity and heat), raw materials and consumables, water (utility and process water), transport (raw materials, intermediates, and finished products), infrastructure inputs (plant construction and annual recurring capital expenditure), purchase of services and indirect goods, employee commuting, and outputs such as wastes (hazardous, non‐hazardous, and aqueous wastes). Regarding spatial aspects, we considered that APIs were expected to be manufactured at 80% in Europe and 20% in the rest of the World. Drugs are expected to be sold in France, but the hypotheses used are valid for Europe in general.

#### Assessed impacts and cutoff

Only GHG emissions were evaluated, as estimation of other environmental impacts is not practically quantifiable by most LCA‐EEIO models, including Exiobase. The impact assessment method used is the Intergovernmental Panel on Climate Change (IPCC) 2021.^18^ No cutoff (ISO 14040‐44) was applied in this study.

#### Estimation of monoclonal antibodies production carbon footprint

**Figure 2:**
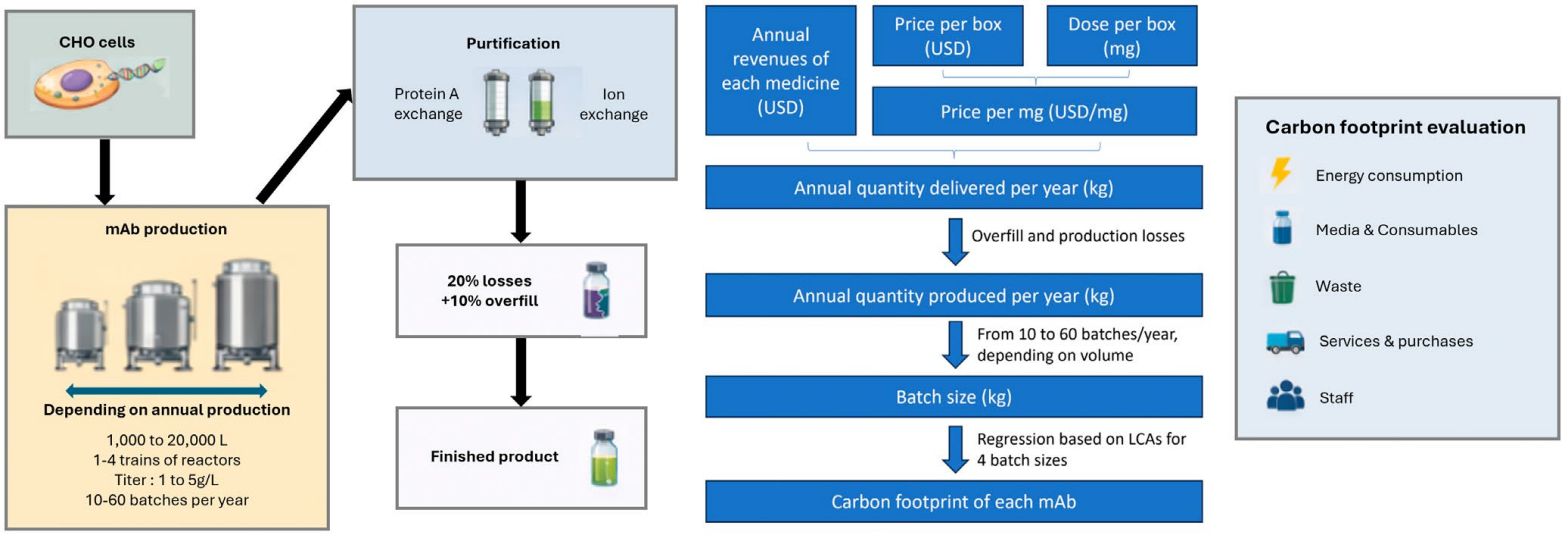
Scheme of the model used to estimate mAb carbon footprints.

Monoclonal antibodies may be produced in bioreactors of different volumes with different titers. We modeled recombinant protein production as when performed using Chinese hamster ovary (CHO) cells, as mAbs are, in the vast majority of cases, produced using these mammalian cells, genetically engineered to express the desired protein. Following transfection, the cells are cultured in bioreactors under sterile, tightly controlled conditions. The carbon footprint of these upstream steps was assessed for several bioreactor sizes (from 1,000 liters up to 20,000 L) and different processes (single‐use technology and stainless‐steel reusable bioreactors). Production yields from 1 g/L (when small volumes are required) to 5 g/L (standard case) were considered, and energy consumption, size of the plant, cell culture media use, consumables use, purchase of services and indirect goods, waste quantities, and employee number were derived from existing technical and economic studies on mAb manufacturing processes.^19^, ^20^, ^21^, ^22^, ^23^, ^24^, ^25^, ^26^, ^27^ Information for the downstream process has also been obtained from these sources, including information on buffer, resins, and other specific items for the purification, since harvested antibodies from cell culture are then purified through chromatography, primarily using protein A affinity and ion exchange columns. The LCA of the downstream steps was performed for four batch sizes, ranging from 0.7 kg to 85 kg of protein. mAb carbon footprint for intermediate‐size batches was obtained using an exponential regression (*r* ^2^ = 0.997). Since the mAb environmental footprint highly depends on the batch size, the annual volume of the 103 mAbs was extrapolated from annual revenues, average prices per dose and strength in mg of protein per dose. A 10% overfill was added to this volume,^28^, ^29^ and also 20% losses all along the process, from mAb manufacturing to fill and finish, packaging, and final quality inspection. Depending on the annual production volume, we considered that 10 (small volume, corresponding to one reactor train for the upstream steps, with an average duration of 36 days per batch, including annual shutdown and inter‐batch cleaning and setup) to 60 (high volume mAbs, corresponding to 4 upstream bioreactor trains, highly optimized with a 24‐day average duration) batches were run annually. This model was used to estimate a batch size, in kg of protein per batch, for each of the 103 mAbs, and then a carbon footprint in kgCO_2_eq/kg. A summary of the process is provided in **Figure** **2**.

### Transport from the drug substance to the fill and finish facility

Transport of the drug substance from its production site to the fill and finish facility in a refrigerated system was estimated to a mean distance of 1,000 km by air transport (0.837 kgCO_2_e/ton/km^30^) and 1,000 km (0.728 kgCO_2_e/ton/km^30^) by road transport and is not specific to a particular production country, leading to an emission factor of 1.57 kgCO_2_e/kg of drug substance.

#### Fill and Finish

The fill and finish facility impact was estimated using a dedicated model based on a surface of 2000 m^2^ to produce between 1 and 10 million doses per year, depending on the annual volume of the medicine, with a mean power consumption of 3,919 kWh/m^2^/year,^31^ divided in 50% natural gas and 50% electricity (we considered that 80% of the electricity was consumed in Europe and 20% globally). Capital goods were estimated to a mean of 20,832 €/m^2^ based on data from 7 manufacturers from press releases^32^, ^33^, ^34^, ^35^, ^36^ with a 50‐year amortization (0.420 kgCO_2_e/€^30^). Capital expenditure was estimated to 2% of capital goods, that is, 417 €/m^2^ (0.340 kgCO_2_e/€^30^). Purchases of goods and services were estimated to represent 50% of capital emissions. The emissions of energy and capital goods were distributed either to 1 million, 6 million or 10 million units produced per year, depending on the number of boxes produced for each medicine.

#### Packaging

Fifteen different drugs were weighted to obtain the mean weight of the vial (10.7 ± 8.1 g, borosilicate glass with an aluminum cap and a rubber seal, cardboard for the box and paper for the leaflet), prefilled syringe (18 ± 12.1 g), auto‐injector (26 ± 3.7 g), secondary packaging (13.1 ± 4.4 g), and leaflet (10 ± 3.2 g). Tertiary packaging was estimated to be similar to secondary packaging. Each of these weights was converted into kgCO_2_e using an adapted Ecoinvent emission factor,^37^ taking into account a 20% loss during production. The carbon footprint of pens and pre‐filled syringes is from a Alastair *et al*.^38^

#### Estimation of wastes and workers commuting

Wastes were accounted for 27 gCO_2_e/unit, based on a waste quantity equal to 20% of the finished medicine weight, including tertiary packaging (55 g). Employees commuting represented 8.3 × 10^−6^ employee/dose (representing 50 employees for the production of 6 million units per year), with an estimated mean distance to get to work of 25 km.

#### Transport from the packaging facility to the pharmacy

Transport of the medicine box from the production facility in a refrigerated system to the pharmacy was estimated to a mean distance of 1,000 km by air transport and 1,000 km by road transport, and is not specific to a particular production country. The medicine box was estimated to represent 50% of the transported weight; the other 50% was expected to be represented by the tertiary packaging, which can include an active or passive refrigerating system.

#### Estimation of corporate carbon footprint

Corporate activities (R&D, sales & marketing, regulatory, general administration, SG&A, etc.) are defined as all activities that are not directly related to the manufacturing and transport of the medicine.^39^ We used results from Piffoux *et al*.,^13^ that used two different approaches to estimate the corporate activities carbon footprint of 24 pharmaceutical companies, either based on companies' carbon accounting (obtained from the Carbon Disclosure Project) or based on an EEIO approach from their financial results combined with Exiobase emission factors. In this work, we used the mean value for innovative pharmaceutical companies, which is estimated to be 0.051 ± 0.011kgCO_2_e/€. This monetary emission factor was applied to the net price perceived by the pharmaceutical company for the box of medicine, by deducting from the regulated price of each box of medicine the wholesaler, pharmacy and tax fraction, which varies depending on the drug price^40^. Overall, it is estimated that 59.7%^41^ of the drug regulated price available in the Agence Nationale de Sécurité du Médicament (ANSM) databases (regulated price from France) is allocated to the pharmaceutical company. In Europe, the average share of the drug price allocated to the pharmaceutical company is similar and estimated at 67%.^42^ This method estimates the SG&A emissions that will be funded by the profit generated by this medicine, to develop future medicines, and is expected to better represent the SG&A footprint of the drug instead of only taking into account R&D expenses related to the development of the drug of interest. Indeed, restricting the scope to the drug of interest implies that drugs whose development was stopped/failed (representing a large part of R&D) are not considered.

#### Estimation of uncertainty

Our model is based on estimates with an associated uncertainty. Confidence intervals surrounding emission factors from Exiobase, ecoinvent, or the French environmental protection agency (ADEME) were considered as 95% confidence intervals (CIs) of normal distributions. All distributions were considered to be normally distributed, and overall uncertainty was estimated using the quadratic sum of uncertainties. CIs and standard deviations were calculated from the distribution obtained for each drug.

### Case applications

#### Comparison of alternative strategies for the second‐line treatment of rheumatoid arthritis with risk factors

We used the mAb database, and the one we recently published on the carbon footprint of oral medicines^13^ to compare the carbon footprint of treatment options in the second line of rheumatoid arthritis, a disease with a prevalence of 0.5–1% in the general population. We estimated the number of administrations and doses in the first year of treatment for a typical patient of 65 kg.

#### Alternative dosing for immunotherapies in oncology

Malmberg *et al*. recently published a study on the impact of alternative dosing strategies (ADSs) for pembrolizumab and nivolumab.^43^ ADSs use reduced doses for patients with smaller weights instead of flat doses. In the absence of alternative proposals, the authors used a mean impact for antibodies of 0.277 kgCO_2_e/mg of antibody. We used our model to re‐estimate the impact of ADSs on treatment carbon footprint using more precise and complete estimations. We added an alternative strategy with a 2‐fold reduction in pembrolizumab “low dose” that is currently investigated in the MOIO trial,^44^ as well as an alternative strategy with “low‐dose” nivolumab (12‐fold decrease) that was recently investigated.^13^ Consultations were considered to be grouped with outpatient hospitalizations, and therefore considered to have a similar and limited impact (about 6 kgCO_2_e/visit from^43^) from one strategy to another.

## RESULTS

### Database description

**Figure 3:**
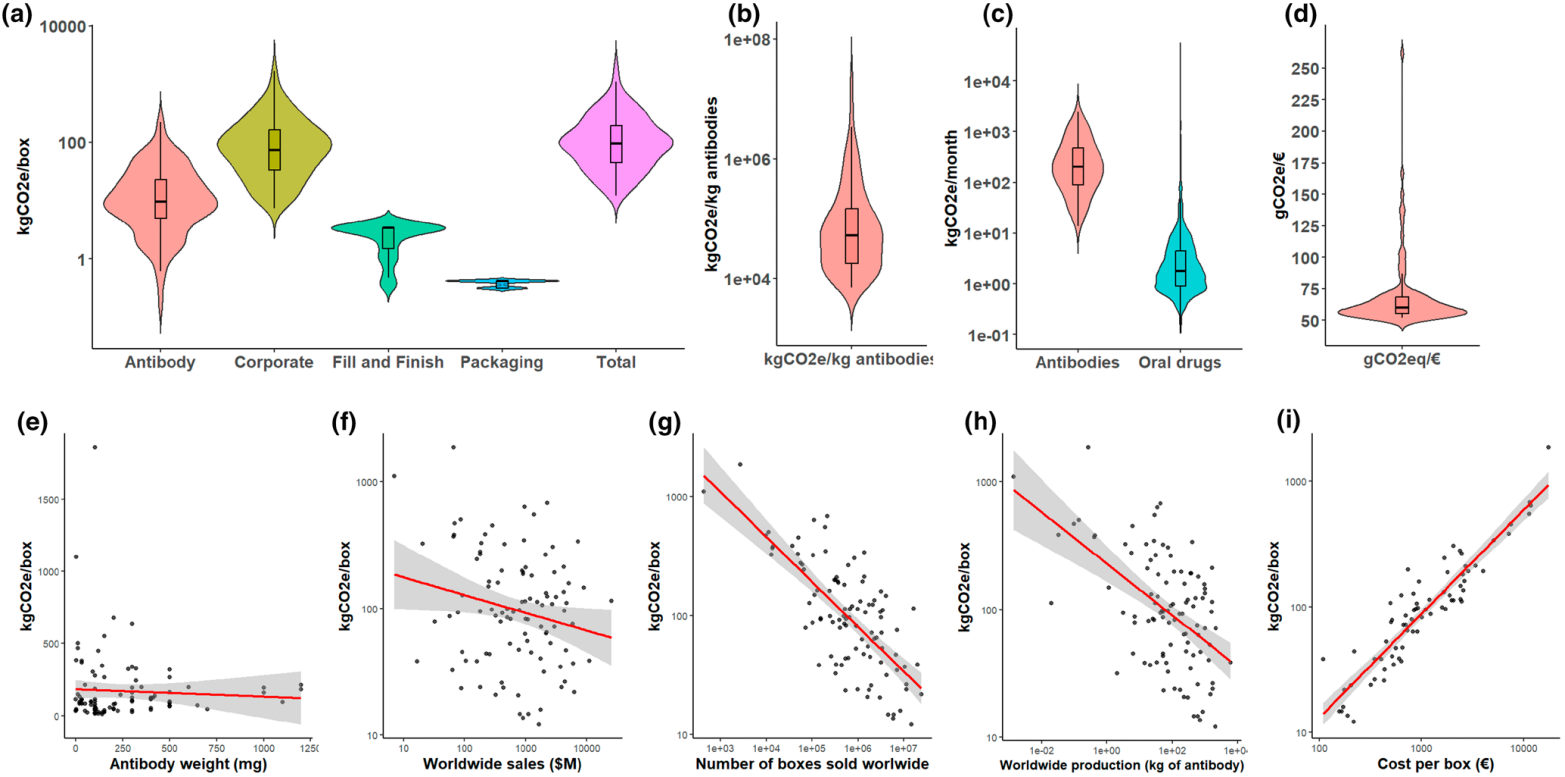
Antibody database description. (**a**) Distribution of monoclonal antibody‐based medicines, carbon footprint, and distribution among its subparts. (**b**) Distribution of monoclonal antibodies production carbon footprint in kgCO_2_e per kg of antibody. (**c**) Distribution of carbon footprint per month of treatment in kgCO_2_e per month for antibody‐based and oral drugs, including 8,182 oral medicines with available defined daily dose.^13^ (**d**) Distribution of monetary emission factor in gCO_2_e/€. (**e**) Relation between the carbon footprint per box and antibody weight/box. (**f**) Relation between the carbon footprint per box and worldwide sales of the product. (**g**) Relation between the carbon footprint per box and the number of boxes sold worldwide. (**h**) Relation between the carbon footprint per box and the estimated worldwide production in kg of antibodies. (**i**) Relation between carbon footprint per box and cost per box in €.

The carbon footprint of mAb‐based medicines is important, representing a mean carbon footprint of 169.7 kgCO_2_e/box (95% CI 10.1–621.1, **Figure** **3** **a** and **Table** **1**). Overall, corporate costs represent 80.4% (35.4–96.9%) of the medicine carbon footprint, while antibody production represents 15.87% (1.3–60.1%) of the total carbon footprint. Overall, fill and finish, and packaging have a much smaller impact of 3.10 and 0.63%, respectively.

**Table 1:** Distribution of the monoclonal antibodies' carbon footprint among subparts

Subpart	Mean fraction (95% CI)	Mean 95% confidence interval (in %)	Mean carbon footprint in kgCO_2_e/month (95% CI)	Mean carbon footprint in kgCO_2_e/box (95% CI)
Corporate	80.4% (35.4–96.9%)	43.04%	300.40 (13.0–1,317)	145.78 (10.1–621.1)
Antibody production	15.87% (1.3–60.1%)	21.56%	61.53 (0.74–323.8)	21.02 (0.7–82.4)
Fill and finish	3.10% (0.4–10.4%)	26.67%	8.13 (0.49–39.23)	2.54 (0.34–3.4)
Packaging	0.63% (0.05–2.70%)	99.98%	1.32 (0.10–5.74)	0.38 (0.31–0.41)
Total	100%	35.18%	371.4 (23.1–1,479)	169.7 (14.6–656.2)

Monoclonal antibodies' production carbon footprint is highly variable depending on the production scale, ranging from 7.1 to 20,206 kgCO_2_e/g of antibody, with a mean of 472 kgCO_2_e/g (95% CI 8.2–2,529 kgCO_2_e/g) (**Figure** **3** **b**).

The 95% CI surrounding the mAb production carbon footprint is estimated to be ±21.56% (**Table** **1**), while the mean complete 95% CI is estimated to be 35.18%.

A month of treatment with a mAb‐based medicine is estimated to be 371.4 kgCO_2_e/month (95% CI 23.1–1,479), whereas the mean treatment emission with an oral treatment is estimated to be 14.1 kgCO_2_e/month (95% CI 0.34–36.2, **Figure** **3** **c**, **Table** **1**).

Monetary emission factor of antibodies is also highly variable, with a mean of 68.9 gCO_2_e/€ (95% CI 52.6–144 gCO_2_e/€, **Figure** **3** **d**). To better understand the effect of production scale and the estimated carbon footprint, we explored the relation between drug carbon footprint per box and worldwide sales in $, number of boxes sold, total production in kg of antibodies and price per box (**Figure** **3** **e–i**). Interestingly, antibody weight per box and worldwide sales (in $) have a limited correlation with medicine boxes' carbon footprint, while the number of boxes sold and total antibody production in kg are better correlated. The best predictor remains the price per box.

### Case applications

#### Comparison of alternative strategies for the second line treatment of rheumatoid arthritis with risk factors

In order to illustrate the interest of the database, we compared the carbon footprint of treatment options that may be offered to patients with rheumatoid arthritis with risk factors in the second line (or more) in combination with methotrexate. All treatment options have a significant carbon footprint, ranging from 286 to 2,047 kgCO_2_e/year (**Table** **2**). Interestingly, the difference may be very important when comparing biotherapies (range 286–2,047 kgCO_2_e/year) and tyrosine kinase inhibitors (range 291–680 kgCO_2_e/year). The difference is also important among biotherapies; rituximab has the lowest carbon footprint of 286 (180–391) kgCO_2_e/year, explained by the need for only two injections, whereas other strategies like TNFα inhibitors are associated with the annual emission of 579–1,802 kgCO_2_e.

**Table 2:** Comparison of alternative strategies for the second line treatment of rheumatoid arthritis with risk factors

	1st year of treatment	Posology	Administration scheme	Annual treatment‐related emissions in kgCO_2_e (95% CI)
TNFα inhibitor	Adalimumab	40 mg/injection	Q2W	978 (770–1,186)
	Certolizumab	200 mg/injection	Q2W, twice the dose at W0, 2 and 4	1,791 (1,078‐2,504)
	Etanercept	50 mg/injection	Weekly	1,802 (1,078‐2,525)
	Golimumab	50 mg/injection	Monthly	1,016 (641–1,391)
	Infliximab	3 mg/kg (65 kg)	W0, W2, W6 weeks (3 mg/kg), then Q8W (5 mg/kg)	403 (341–464)
Anti‐IL‐6	Tocilizumab	162 mg/injection	Weekly	928 (786–1,070)
	Sarilumab	200 mg/injection	Q2W	2,047 (1,355‐2,738)
Anti‐CD‐20	Rituximab	1,000 mg/injection	2 injections per year	286 (180–391)
T‐cell costimulation inhibitor	Abatacept	125 mg/injection	Weekly	822 (538–1,106)
JAK inhibitor	Baricitinib	4 mg/day PO	QD	294 (176–411)
	Filgotinib	200 mg/day PO	QD	680 (293–1,068)
	Tofacitinib	5 mg × 2/day PO	BID	360 (223–497)
	Upadacitinib	15 mg/day PO	QD	418 (251–585)

#### Alternative and low dosing for immunotherapies in oncology

We used the database to investigate the effect of ADSs of pembrolizumab and nivolumab in oncology. We used our model to update the article from Malmberg *et al*.^43^ that investigated the carbon footprint of these ADSs, using a more precise carbon footprint. Interestingly, ADSs are associated with a decrease of 7–22 kgCO_2_e per week of treatment (from −9.5 to 28.2%, **Table** **3**). The absolute emissions estimated per week are 2‐ to 4‐fold higher than those proposed by Malmberg *et al*.,^43^ but the relative amount (%) of emission mitigations due to ADS is very similar. We also provide data on another ADS that is currently still under study, namely “low‐dose” immunotherapies, which imply decreasing pembrolizumab doses by 2‐fold,^44^ and 12‐fold for nivolumab.^13^ These strategies are associated with a decrease of 38.3 kgCO_2_e per week of treatment for pembrolizumab (−48.7%), and 69.7 kgCO_2_e per week of treatment for nivolumab (−91.7%).

**Table 3:** Effect of alternative and low‐dosing strategies on the carbon footprint of cancer immunotherapy

		Energy use^a^	Patient travel^a^	Staff travel^a^	Medical equipment^a^	Waste^a^	Pharmaceutical distribution^a^	Pharmaceutical manufacturing using the full LCA (vs. from Malmberg *et al*.^a^)	Total (vs. from Malmberg *et al*.^a^)	Total per week of treatment (vs. from Malmberg *et al*.^a^)	kgCO_2_e reduction by ADS per week (%)	kgCO_2_e reduction by changing dose interval per week (%)	Total kgCO_2_e reduction with ADS‐max interval per week (%)	kgCO_2_e reduction with “Low dose” reduction per week (%)
Pembrolizumab	200 mg Q3W	1.11	2.34	0.1	1.13	1.62	0.02	**229.5 (55.4)**	**235.9 (61.7)**	78.6 (20.6)				
	ADS Q3W	1.11	2.34	0.1	1.08	1.53	0.01	**163.3 (39.4)**	**169.4 (45.6)**	56.5 (15.2)	−22.1 (−28.2%)			
	400 mg Q6W	1.11	2.34	0.1	1.32	1.88	0.04	**459.1 (110.8)**	**465.9 (117.6)**	77.6 (19.6)		−1.0 (−1.2%)		
	ADS Q6W	1.11	2.34	0.1	1.23	1.75	0.03	**357.2 (86.2)**	**363.7 (92.8)**	60.6 (15.5)	−17.0 (−21.7%)	4.1 (7.3%)	−18.0 (−22.9%)	
	« Low dose » 200 mg Q6W (hypothesis)	1.11	2.34	0.1	1.13	1.62	0.02	**114.8 (27.7)**	**121.1 (34.0)**	40.4 (11.3)				−38.3 (−48.7%)
Nivolumab	240 mg Q2W	1.11	2.34	0.1	1.1	1.64	0.05	**145.6 (66.5)**	**152.0 (72.8)**	76.0 (36.4)				
	Q2W ADS	1.11	2.34	0.1	1.18	1.7	0.07	**131.0 (59.8)**	**137.5 (66.3)**	68.7 (33.1)	−7.3 (−9.5%)			
	360 mg Q3W	1.11	2.34	0.1	1.26	1.88	0.1	**218.5 (99.7)**	**225.2 (106.5)**	75.1 (35.5)		−0.9 (−1.2%)		
	Q3W ADS	1.11	2.34	0.1	1.28	1.86	0.11	**194.1 (88.6)**	**200.9 (95.4)**	67.0 (31.8)	−8.1 (−10.7%)		−9.0 (−12.0%)	
	480 mg Q4W	1.11	2.34	0.1	1.23	1.87	0.1	**291.3 (133.0)**	**298.0 (139.8)**	74.5 (34.9)		−1.5 (−2.0%)		
	Q4W ADS	1.11	2.34	0.1	1.31	1.93	0.12	**258.2 (117.9)**	**265.1 (124.8)**	66.3 (31.1)	−8.2 (−10.8%)		−9.7 (−13.0%)	
	« Low‐dose » 20 mg Q3W (hypothesis)	1.11	2.34	0.1	1.28	1.86	0.11	**12.1 (5.5)**	**18.9 (12.3)**	6.3 (4.1)				−69.7 (−91.7%)

*Note:* Bold values represent carbon footprint, unit: kgCO_2_e, e.g., 229.5 (55.4) = >229.5 kgCO_2_e in our estimation versus 55.4 kgCO_2_e as estimated by Malmberg et al. ADS, alternative dosing strategies.

^a^ Denotes data from Malmberg *et al*.^43^

## DISCUSSION

We describe for the first time an estimation of the carbon footprint of mAb‐based medicines, in open access for health professionals. This database provides the carbon footprint from cradle to gate of the pharmacy, with all life cycle stages considered, including manufacturing and transport of all ingredients, fill and finish, packaging and corporate activities (Sales, General & Administration, R&D). The database also provides for each medicine an estimation of uncertainty surrounding the point estimate. Our estimates do not include (i) emissions from the pharmacy and hospital, (ii) patient travel to the pharmacy or hospital, and (iii) medicine end of life. Compared to the cradle‐to‐grave medicine carbon footprint, GHG emissions related to the preparation of the medicine by the pharmacy are expected to be very limited, from 20–120 gCO_2_e/injection as estimated by Malmberg *et al*. in the Netherlands^43^ up to 3–4 kgCO_2_e/injection or infusion for cytotoxic mAbs [unpublished results]. Waste management is also estimated to be limited, in the 1.53–1.93 kgCO_2_e/injection.^43^ Patient travel to the pharmacy is expected to be in the range of 0.26 kgCO2e per box of medicine sold^45^ and may be a little bit longer to get to the hospital, estimated at 2.34 kgCO_2_e for Malmberg *et al*. in the Netherlands^43^ and 6–7 kgCO2e in our experience for immunotherapies (unpublished data).

Our overall estimates may hardly be compared to other results in the literature in the absence of examples that disclose the *complete* carbon footprint of mAb‐based medicine, in the absence of other LCAs detailing the complete carbon footprint of these drugs.

Other authors usually focused on mAb production, without taking into account transport, packaging, fill and finish, and more importantly, corporate activities. Amasawa *et al*.^46^ published an estimate restricted to nivolumab production carbon footprint, estimated to 177–277 kgCO_2_e/g of antibody depending on the use of single‐use or re‐usable bioreactors, but did not consider the downstream steps.

Budzinski *et al*. published another estimation of 22.7 kgCO_2_e/g of antibody produced in single‐use bioreactors, a 10‐fold lower estimate that does not take into account capital goods, material transport, losses, waste, employees' transport, impact of different production scales, and purification yield.^47^ The U.S. EEIO provides an emission factor for vaccines and biological products (87 gCO2e/$),^7^ that is in the range of emission factors we obtain in our analysis (mean value of 68.9 gCO_2_e/€, 95% CI 52.6–144 gCO_2_e/€).

We demonstrate in this study that the production of antibodies has a largely variable carbon footprint that largely depends on the production scale/bioreactor size. The use of a single emission factor for all antibodies does not take into account this important effect, with a > 1,000‐fold variation.

None of the previous studies has taken into account the effect of corporate costs on antibody‐based medicines. As for our previous studies in oral medicines,^13^ we allocated corporate costs linearly with drug price, implying for high‐cost drugs like antibody‐based drugs an important impact of corporate costs, representing a mean of 80% of the total carbon footprint for these drugs. The hypothesis of a linear relation between cost per box and corporate footprint is supported by the fact that high‐cost medicines require large R&D investments (fundamental research, clinical trials, etc.), and are associated with a larger sales workforce per box. Lastly, a large fraction of sales from innovative pharmaceutical companies is represented by high‐cost drugs, which are therefore not exceptions in their medicine portfolio. Our analysis is focused on the carbon footprint of medicines, as the estimation of other pollutants is difficult to perform in the absence of emission factors for these pollutants. The main impact of the geographical scope is on electricity, since natural gas (considered to produce steam) has the same impact worldwide. Country‐to‐country differences related to antibody production, transport, and packaging are expected to have a limited impact and represent a small fraction of the carbon footprint. Although based on the French pharmacopeia, the results are therefore valid in the European context. Countries with the lowest mix grid electricity emission factor, in kgCO_2_e/kWh, enable a lower carbon footprint production since electricity‐related emissions are a major contributor to the API footprint (about 27–33% of the API footprint) in our model. Single‐use technologies were associated with an 8%–11% lower carbon footprint, explained by a lower energy consumption, smaller manufacturing infrastructure and higher productivity per employee, even if single‐use technology is consuming much more consumables than reusable bioreactors. The corporate emission factor is estimated from international data in a globalized industry, and therefore adapted in every country. In our model, the carbon footprint is proportional (although not linearly) to the batch size. Therefore, apart from uncertainties regarding emission factors that are taken into account in the uncertainty analysis, the main limitation is the modeling of the batch size through annual revenues, used to approximate the annual production volume. However, the total volume of production for most, if not all, biotherapies is not publicly available.

Our case applications aim at showing how this database may be used. We show that compared to oral medicine‐based care pathways, biotherapy‐based care pathways tend to be associated with a larger carbon footprint. This is the case for JAK inhibitors compared to most other biotherapies options used in second line (or more) of treatment in rheumatoid arthritis. Of note, rituximab biotherapy remains the treatment modality that has the lowest carbon footprint, even compared to JAK inhibitors, due to its remanent effect, only requiring two injections for about a year of treatment. We think that the carbon footprint of drugs may also be a variable to take into account when choosing a treatment, as well as patient characteristics, adverse effects, treatment costs, or injection route.

In oncology, ADSs for immunotherapies allow for mitigating the very large impact of immunotherapies (estimated to be about 4 tCO_2_e/year) by 10–28%. More importantly, “low‐dose” strategies may mitigate GHG emissions by up to 92% for low‐dose nivolumab if these strategies were demonstrated to be non‐inferior. Of note, these case applications are only illustrative, and cannot be the only variable to take into account when choosing a treatment strategy.

We provide the first open access database detailing the carbon footprint of mAbs and related biotherapies. We show mAb‐based drugs have a large GHG footprint, among which a large fraction is emerging from corporate emissions. We hope this database will help to better eco‐design care pathways and inform health professionals' decisions, who often lack environmental data regarding their prescriptions. This work is part of a larger project that aims to provide the carbon footprint of the full *pharmacopeia* on our website (www.ecovamed.com).

## FUNDING

The monoclonal antibody carbon footprint modeling is part of the LIBRA project (Light Based Multisensing Device for Screening of Pathogens and Nutrients in Bioreactors), which was co‐funded by the Horizon Europe research and innovation program, under grant agreement 101093150.

## CONFLICT OF INTEREST

ST and MF are both working at Ecovamed and ST has shares in Ecovamed (Founder). All other authors declared no competing interests for this work.

## AUTHOR CONTRIBUTIONS

S.T., M.F., M.S., and M.P. wrote the manuscript. S.T., M.F., and M.P. designed the research. S.T., M.F., M.S., and M.P. performed the research. S.T., M.F., and M.P. analyzed the data.

## DECLARATION OF GENERATIVE AI AND AI‐ASSISTED TECHNOLOGIES IN THE WRITING PROCESS

No AI tool was used to produce this article.

## Data Availability

Medicines' detailed carbon footprints are available on http://www.app.ecovamed.com. Detailed data are available to health professionals for research purposes with a signed data access agreement upon request to sebastien.taillemite@ecovamed.com.
